# Ferroptosis: biology and role in liver disease

**DOI:** 10.1007/s00535-025-02300-5

**Published:** 2025-09-18

**Authors:** Keisuke Hino, Sohji Nishina, Izumi Yanatori

**Affiliations:** 1 Digestive Disease Center, Shunan Memorial Hospital, 1-10-1 Ikunoya-Minami, Kudamatsu, Yamaguchi 744-0033 Japan; 2 Department of Molecular and Cellular Physiology, Graduate School of Medicine, Kyoto University, Kyoto, 606-8501 Japan; 3 Department of Gastroenterology and Hepatology, Kawasaki Medical School, Kurashiki, Japan

**Keywords:** Iron, Glutathione peroxidase 4, Lipid peroxidation, Polyunsaturated free fatty acid, Reactive oxygen species

## Abstract

Ferroptosis is a form of nonapoptotic cell death that is driven by iron-dependent lipid peroxidation and is relevant to a wide range of biological processes, such as development, aging, immunity, and cancer. Ferroptosis has also been linked to numerous hepatic metabolic pathways, including the metabolism of iron, fatty acids, and amino acids, such as cysteine. During the last decade, studies on the biology of and molecules regulating ferroptosis have shed light on the role of ferroptosis in liver disease and its implications. The susceptibility of liver cells to ferroptosis determines the extent of liver injury and affects the progression of nonneoplastic diseases, whereas liver cancer cells display intrinsic or acquired resistance to ferroptosis, which promotes cancer progression. These findings indicate that ferroptosis represents a promising target for the prevention and treatment of many forms of liver disease. In this review, we provide an update on the mechanisms regulating ferroptosis, focusing on the peroxidation of phospholipids, the antioxidant pathways that limit lipid peroxidation, and the regulation of the labile iron pool, all of which are closely connected. We also summarize the roles and importance of ferroptosis in the pathogenesis of liver disease, and the therapeutic potential of targeting ferroptosis in liver diseases.

## Introduction

Ferroptosis is an iron-dependent programmed cell death mechanism that is distinct from apoptosis and other forms of cell death. Classical features of apoptosis, such as mitochondrial cytochrome c release, caspase activation, and chromatin fragmentation, are not observed in ferroptosis. It is driven by the peroxidation of phospholipids (PLs), which is triggered by the accumulation of reactive oxygen species (ROS) [1, 2]. PL peroxidation compromises the integrity of plasma membranes, leading to cell damage and the release of damage-associated molecular patterns (DAMPs) [1].

The liver is the central organ for systemic iron storage and regulation, lipid metabolism, and detoxification. Ferroptosis has also been linked to numerous hepatic metabolic pathways, including those involved in the metabolism of iron, fatty acids (FAs), and amino acids such as cysteine [3]. These physiological characteristics suggest a tight link between hepatic homeostasis and ferroptotic signaling. The susceptibility of liver cells to ferroptosis determines the extent of liver injury and affects the progression of nonneoplastic diseases, and liver cancer cells can display intrinsic or acquired resistance to ferroptosis [4]. These findings imply that ferroptosis represents a promising target for the prevention or treatment of many forms of liver disease.

Notably, as of June 19, 2025, a PubMed search Yielded 1,940 research publications on ferroptosis in the liver, which implies that the liver ranks second, after the brain (1,997 publications), as the most studied organ in terms of ferroptosis. This highlights the intense research activity in this area and the need to synthesize and update the growing body of literature. In this review, we aim to provide a comprehensive overview of recent advances in our understanding of the molecular mechanisms and regulatory pathways of ferroptosis, and its roles in the pathogenesis and progression of liver diseases.

## Mechanisms and regulation of ferroptosis

### Phospholipid metabolism involved in the regulation of ferroptosis (Fig. 1)

**Fig. 1:**
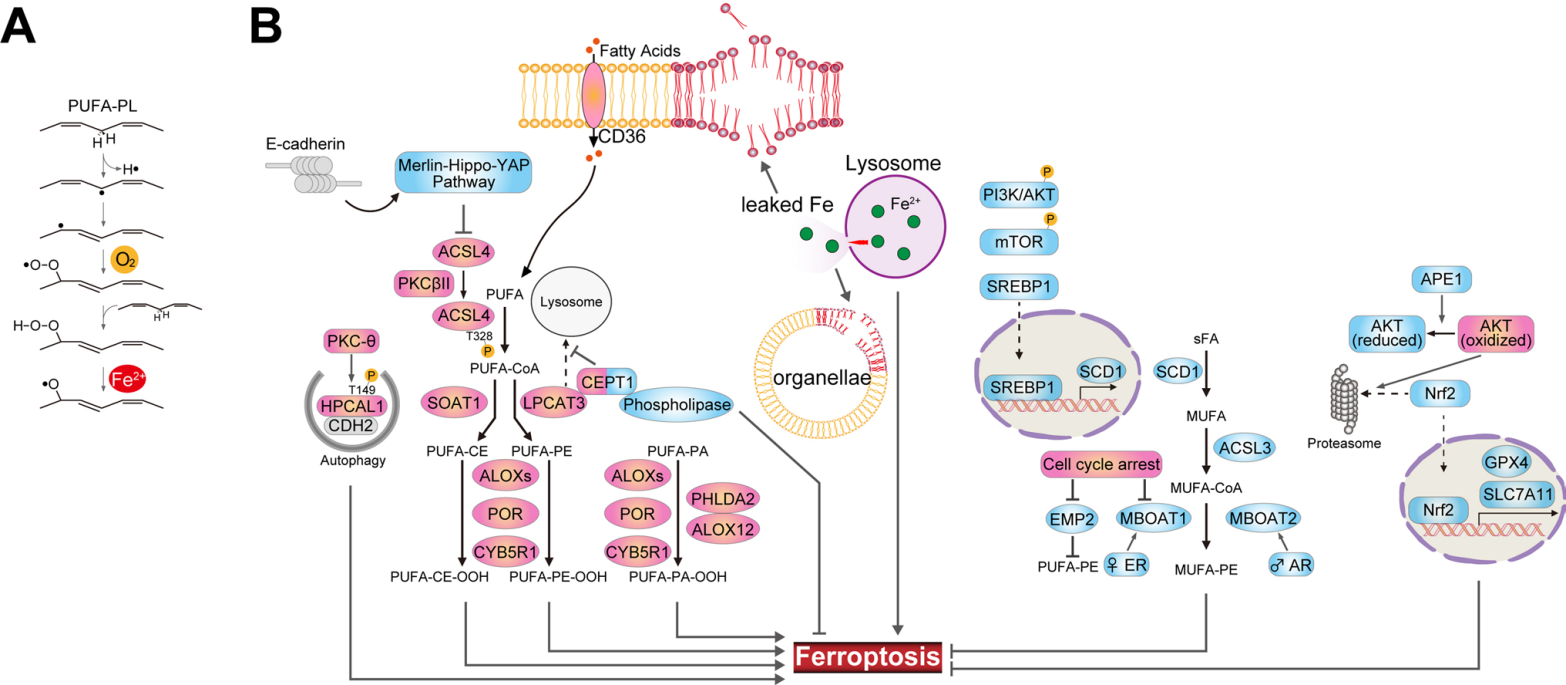
Phospholipid metabolism involved in the regulation of ferroptosis. A Lipid peroxidation chain reaction. B Polyunsaturated fatty acids are highly susceptible to peroxidation, and through various signaling pathways, this increases susceptibility to ferroptosis. In contrast, high monounsaturated fatty acid concentrations confer resistance to ferroptosis by reducing lipid peroxidation. The molecules, signaling pathways, and cellular conditions shown in red are proferroptotic factors, and those in blue are antiferroptotic factors. Each of the pathways that regulate ferroptosis is explained in detail in the text. *ACSL4* acyl-coenzyme A synthetase long-chain family member 4, *AKT* protein kinase B, *ALOX* arachidonate lipoxygenase, *APE1* apurinic/apyrimidinic endonuclease 1, *AR* androgen receptor, *CDH2*, cadherin 2, *CEPT1* choline/ethanolamine phosphotransferase 1, *CYB5R1* cytochrome b5 reductase 1, *EMP2* epithelial membrane protein 2, *ER* estrogen receptor, *GPX4* glutathione peroxidase 4, *HPCAL1* hippocalcin-like 1, *LPCAT3* lysophosphatidylcholine acyltransferase 3, *MBOAT1* membrane-bound O-acyltransferase domain-containing 1, *mTOR* mammalian target of rapamycin, *Nrf2* nuclear factor erythroid 2-related factor 2, *PHLDA2* pleckstrin homology-like domain family A member 2, *PI3K* phosphatidylinositol 3-kinase, *PKCβII* protein kinase C beta type isoform 2, *PKCθ* protein kinase C theta, *PL* phospholipid, *POR* P450 oxidoreductase, *PUFA* polyunsaturated fatty acid, *SCD1* stearoyl-CoA desaturase, *SLC7A11* solute carrier family 7 member 11, *SOAT1* sterol O-acyltransferase 1, *SREBP1* sterol element-binding protein 1, *YAP* Yes-associated protein

#### PLs and polyunsaturated fatty acids (PUFAs)

Lipid peroxidation involves the replacement of a hydrogen atom attached to a carbon atom with a peroxyl group (O–O), which suggests that the susceptibility of a lipid to peroxidation depends on the strength of its carbon–hydrogen (C–H) bonds [5]. PUFAs are particularly susceptible to peroxidation, owing to the extremely weak C–H bonds between adjacent C = C double bonds, which makes them key substrates for ferroptosis [5]. A very recent study demonstrated that the intralysosomal lipid peroxidation reaction occurs during the initiation of ferroptosis, and triggers iron leakage, fostering a cell-wide lipid peroxidation reaction by increasing lysosomal membrane permeabilization [6].

The de novo synthesis and remodeling of PLs regulate sensitivity to ferroptosis, because they determine the composition of the plasma membrane [7]. Acyl-coenzyme A (CoA) synthetase long-chain family member 4 (ACSL4) and lysophosphatidylcholine acyltransferase 3 (LPCAT3) activate and incorporate PUFAs into membrane-bound lipids [8, 9]. ACSL4 preferentially acylates arachidonic acid, and LPCAT3 inserts acylated arachidonic acid into membrane PLs [9]. ACSL4 is involved in two downstream pathways that yield different PUFA-related acyl-CoA esters. One involves the incorporation of PUFAs into phosphatidylethanolamines (PEs) by LPCAT3 [8–10], and the other generates PUFA-cholesterol esters through the action of sterol O-acyltransferase 1 [11]. The phosphorylation of ACSL4 at Thr328 by protein kinase C beta type isoform 2 promotes the incorporation of PUFAs into PLs and drives ferroptosis [12].

Cell–cell contacts affect the expression of ACSL4 through the Merlin–Hippo–Yes-associated protein (YAP) pathway, such that its expression is downregulated as the cell density increases and is upregulated by the depletion of E-cadherin [13]. The autophagic receptor hippocalcin-like 1 (HPCAL1) selectively degrades cadherin 2, thereby increasing cellular susceptibility to ferroptosis. This autophagic process requires protein kinase C theta-mediated phosphorylation of HPCAL1 at Thr149 [14]. Choline/ethanolamine phosphotransferase 1 (CEPT1) suppresses ferroptosis, potentially by interacting with phospholipases and metabolizing certain proferroptotic PUFA-containing PLs [15], even though CEPT1 prevents LPCAT3 from undergoing lysosomal degradation.

ACSL4-independent pathways for PUFA peroxidation have also been reported to induce ferroptosis. One pathway includes the pleckstrin homology-like domain family A member 2/arachidonate lipoxygenase 12 (ALOX12) complex and is activated endogenously in vivo as part of tumor suppression [16]. ALOXs, including ALOX12, catalyze PUFA oxygenation and initiate lipid peroxidation by introducing hydroperoxy groups (–OOH) into FA chains [17]. The second inducer of ferroptosis is cytochrome P450 enzymes, which catalyze lipid peroxidation by introducing oxygen atoms into FA chains. Nicotinamide adenine dinucleotide phosphate (NADPH)-cytochrome P450 oxidoreductase (POR) and NADPH-cytochrome b5 reductase 1 transfer electrons from NAD(P)H to oxygen to generate hydrogen peroxide (H_2_O_2_), which subsequently reacts with iron to generate reactive hydroxy radicals (the Fenton reaction), causing peroxidation of the PUFA chains of membrane PLs [18].

### Monounsaturated fatty acids (MUFAs)

In contrast to PUFAs, both exogenous [19] and de novo synthesized MUFAs [20] inhibit ferroptosis. Specifically, ACSL3-dependent MUFA activation creates a ferroptosis-resistant state by competitively inhibiting PUFA peroxidation, and exogenous MUFAs also protect cells from apoptotic lipotoxicity caused by the accumulation of saturated FAs but in an ACSL3-independent manner [19]. Oncogenic activation of phosphatidylinositol 3-kinase (PI3K)–protein kinase B (AKT)–mammalian target of rapamycin (mTOR) signaling pathway suppresses ferroptosis in cancer cells via sterol regulatory element-binding protein 1 (SREBP1)-mediated production of MUFAs [21]. Membrane-bound O-acyltransferase domain-containing 1 (MBOAT1) and MBOAT2, which are lyso-PL acyltransferases, selectively transfer MUFAs to lyso-PE, thereby increasing cellular MUFA-PE and reducing PUFA-PE concentrations, which results in the inhibition of ferroptosis [22]. Importantly, MBOAT1 and MBOAT2 expression is directly upregulated by estrogen receptor and androgen receptor, respectively, thereby preventing ferroptosis independently of glutathione peroxidase 4 (GPX4) and ferroptosis suppressor protein 1 (FSP1) through a PL remodeling-mediated surveillance mechanism [22]. MBOAT1 also contributes to the cell cycle arrest-associated sensitization of cancer cells to ferroptosis. Lowered expression of MBOAT1 and epithelial membrane protein 2 induces an increase in the abundance of PUFA-PLs upon cell cycle arrest, thereby sensitizing cancer cells to ferroptosis [23].

## Regulation of ferroptosis by ROS (Fig. 2)

**Fig. 2:**
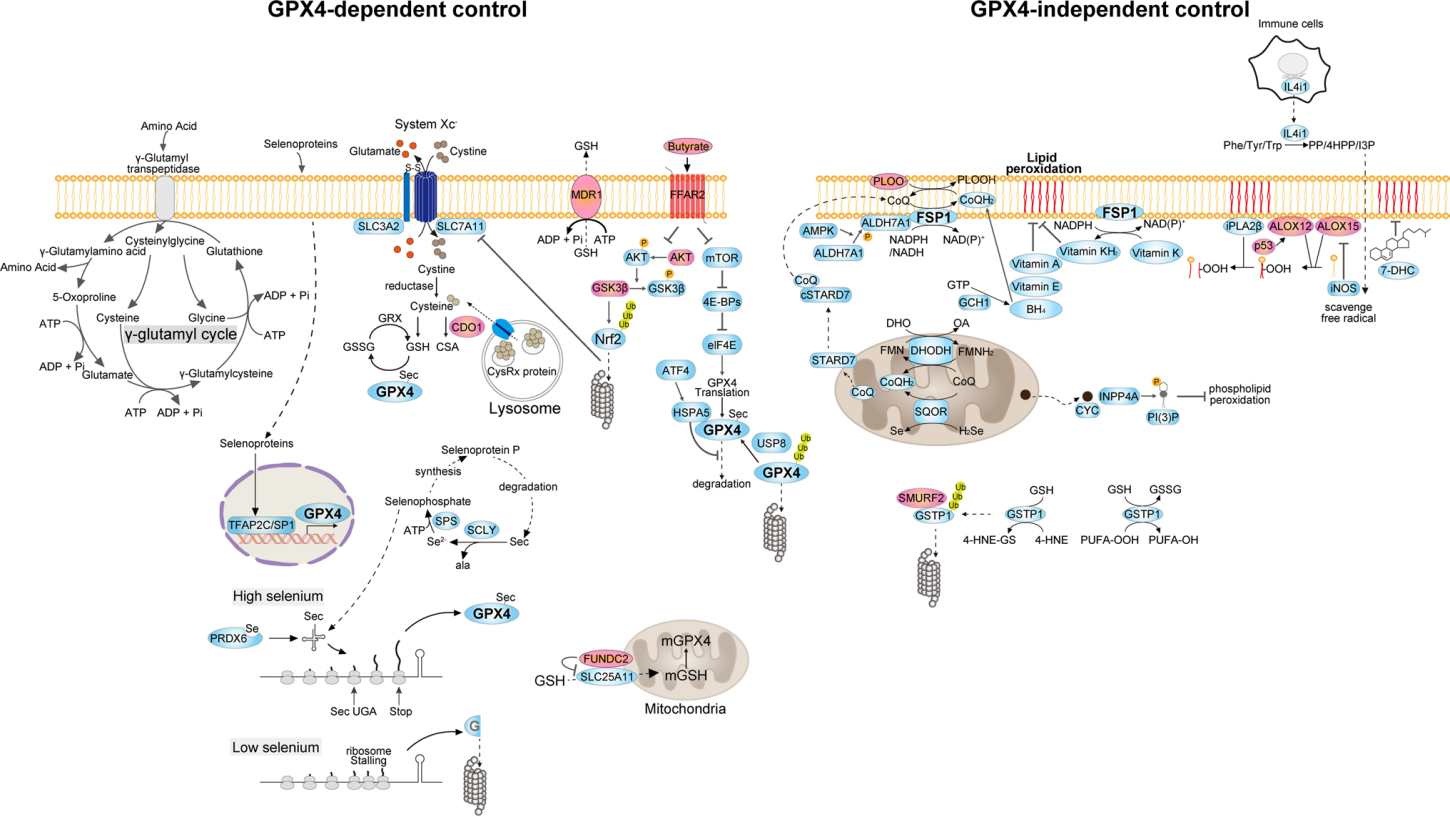
Antioxidant pathways regulating ferroptosis that are dependent on or independent of GPX4. GPX4 plays a pivotal role in the suppression of ferroptosis by neutralizing lipid peroxides. In addition, there are multiple antioxidant pathways that suppress ferroptosis independent of GPX4, which highlights the complex interplay of mechanisms that suppress ferroptosis. Red indicates proferroptotic factors, and blue indicates antiferroptotic ones. Each of the pathways that regulate ferroptosis is explained in detail in the text. *AKT* protein kinase B, *ALDH7A1* aldehyde dehydrogenase 7A1, *AMPK* AMP-activated protein kinase, *ATF4* activating transcription factor 4, *BH*_*4*_ tetrahydrobiopterin, *CDO1* cysteine dioxygenase 1, *CoQ* coenzyme Q, *CoQH*_*2*_ reduced coenzyme Q, *CSA* cysteine sulfinic acid, *CYC* cytochrome *c*, *CysRx* extracellular cysteine-rich, *DHO* dihydroorotate, *DHODH* dihydroorotate dehydrogenase, *eIF4E* eukaryotic translation initiation factor 4E, *FFAR2* free fatty acid receptor 2, *FMN* flavin mononucleotide, *FMNH*_*2*_ reduced flavin mononucleotide, *FSP1* ferroptosis suppressor protein 1, *GCH1* GTP cyclohydrolase 1, *GPX4* glutathione peroxidase 4, *GRX* glutaredoxin, *GSH* glutathione, *GSK3β* glycogen synthase kinase-3 beta, *GSSG* glutathione disulfide, *GSTP1* GSH *S*-transferase P1, *GTP* guanosine triphosphate, *H*_*2*_*Se* hydrogen selenide, *HSPA5* Heat shock 70-kDa protein 5, *IL4i1* Interleukin-4-induced-1, *iNOS* nitric oxide synthetase 2, *INPP4A* inositol polyphosphate-4-phosphatase type IA, *iPLA2β* calcium-independent phospholipase A2β, *I3P* indole-3-pyruvate, *MDR1* Multidrug resistance protein 1, *mTOR* mammalian target of rapamycin, *NADPH* nicotinamide adenine dinucleotide phosphate, *Nrf2* nuclear factor erythroid 2-related factor 2, *OA* orotate, *PI(3)P* phosphatidylinositol-3-phosphate, *PLOO•* phospholipid peroxyradical, *PLOOH* phospholipid hydroperoxide, *PP* phenylpyruvate, *PRDX6* peroxiredoxin 6, *Se* selenium, *Sec* selenocysteine, *SLC3A2* solute carrier family 3 member 2, *SLC7A11* solute carrier family 7 member 11, *SMURF2* SMAD-specific E3 ubiquitin protein Ligase 2, *SP1* specificity protein 1, *SQOR* sulfide quinone oxidoreductase, *STARD7* StAR-related Lipid transfer domain-containing 7, *TFAP2C* transcription factors like transcription factor AP-2 gamma, *USP8* ubiquitin-specific protease 8, *4E-BP* 4E-binding protein, *4HNE* 4-hydroxynonenal, *4-HNE-GS* glutathione S conjugate of 4-HNE, *4HPP* 4-hydroxy-phenylpyruvate

### GPX4-dependent regulation

GPX4 is the primary enzyme that protects against ferroptosis by reducing lipid hydroperoxides in membranes to alcohols [24]. Cysteine is a fundamental constituent of the glutathione (GSH) tripeptide that is required for GPX4 synthesis. System X_c_^−^ is a disulfide-linked Heterodimer that is composed of solute carrier family 7 member 11 (SLC7A11) (xCT) and SLC3A2 and imports cystine in exchange for intracellular glutamate [25]. Once inside the cell, cystine is reduced to cysteine, fueling GSH production. System X_c_^−^ is critical for maintaining intracellular GSH levels, as the enzymatic synthesis of GSH is very slow during the onset of ferroptosis [17]. Cysteine can also be acquired indirectly via the endocytosis of extracellular cysteine-rich proteins, followed by lysosomal catabolism [26]. Extracellular GSH can directly fuel de novo intracellular GSH synthesis via the γ-glutamyl cycle [27]. Activating transcription factor 4 (ATF4)-induced Heat shock 70-kDa protein 5 protects GPX4 from degradation, thereby preventing lipid peroxidation [28]. Furthermore, ubiquitin-specific protease 8 deubiquitinates GPX4, thereby protecting it against proteasome-mediated degradation [29].

In addition to erastin and RAS-selective lethal compound 3 (RSL3), sodium butyrate also reduces SLC7A11 and GPX4 expression by inhibiting the mTOR pathway [30]. The free fatty acid receptor 2 (FFAR2)–AKT–nuclear factor erythroid 2-related factor 2 (Nrf2) and FFAR2–mTOR axes mediate the sodium butyrate-induced downregulation of SLC7A11 and GPX4, respectively. Because GPX4 has a selenocysteine residue (U46) in its active site that is responsible for its hydroperoxidase activity [31], the modulation of selenium/selenocysteine metabolism is emerging as a possible strategy to control ferroptosis [32]. Low selenium levels result in ribosome stalling at the inefficiently decoded GPX4 selenocysteine UGA codon, leading to ribosome collision, early termination of translation, and proteasomal clearance of the N-terminal GPX4 fragment [32]. Transcription factors such as the transcription factor AP-2 gamma and specificity protein 1 are activated by selenoproteins and increase GPX4 expression [33]. Selenocysteine released from selenoprotein P is metabolized by selenocysteine lyase (SCLY), producing selenide which provides selenophosphate for the biosynthesis of Sec tRNA [34]. An alternative pathway involving peroxiredoxin 6 (PRDX6), which is independent of SCLY, increases selenocysteine utilization and maintains GPX4 expression, leading to ferroptosis resistance [35, 36], since PRDX6 acts as a selenium delivery system, facilitating the efficient synthesis of selenocysteyl-tRNA^[Ser]Sec^ [36].

Other molecules that promote ferroptosis include multidrug resistance protein 1 (MDR1) which drives GSH efflux [37], and cysteine dioxygenase 1, which depletes cysteine and GSH [38]. The mitochondrial outer membrane protein FUN14 domain-containing 2 destabilizes SLC25A11, the mitochondrial GSH transporter, and reduces mitochondrial GPX4, resulting in increased ferroptosis [39].

### GPX4-independent regulation

#### FSP1 and coenzyme Q_10_ (CoQ_10_)

FSP1 regulates the reduced form of CoQ_10_ with NADPH, and reduced CoQ_10_ scavenges peroxidized lipids. CoQ_10_ localizes not only to the mitochondria but also to membranes throughout cells, and StAR-related lipid transfer domain-containing 7 facilitates CoQ_10_ transport to the plasma membrane [40]. FSP1 also suppresses ferroptosis by reducing vitamin K to its hydroquinone, which is a potent radical-trapping antioxidant that inhibits lipid peroxidation [41]. AMP-activated protein kinase (AMPK)-mediated phosphorylation of aldehyde dehydrogenase 7A1 recruits FSP1 to cell membranes. This mechanism implies that FSP1 is acutely activated by ferroptotic stress through signaling, rather than operating constitutively [42].

Other endogenous metabolites also serve as radical-trapping antioxidants that inhibit ferroptosis, including tetrahydrobiopterin (BH_4_) [43], vitamin E, and vitamin A [44]. GTP cyclohydrolase 1 suppresses ferroptosis by generating the lipophilic antioxidant BH_4_, which prevents lipid peroxidation by remodeling the lipid membrane environment to increase the content of reduced CoQ_10_ and reduce the concentration of PUFA-PLs [43]. The metabolite of the cholesterol biosynthesis pathway, 7-dehydrocholesterol (7-DHC), is primarily oxidized instead of membrane phospholipids as a radical-trapping agent, and suppresses ferroptosis [45]. Collectively, these pathways rely on NADPH as the electron donor to regenerate radical-trapping antioxidants, highlighting the cellular NADPH pool as a key determinant of ferroptosis sensitivity.

#### Mitochondrial proteins

Like FSP1 in the extramitochondrial membrane, dihydroorotate dehydrogenase, a mitochondrial enzyme involved in pyrimidine biosynthesis, suppresses ferroptosis in the mitochondrial inner membrane by reducing ubiquinone to ubiquinol [46]. Selenium reduces ubiquinone in mitochondria by activating sulfide quinone oxidoreductase, thereby protecting against lipid peroxidation and ferroptosis more rapidly than the mechanism involving selenoprotein production [47].

Cytochrome *c* is a heme protein that is essential for the mitochondrial electron transport chain and pivotal in apoptosis. Recently, an unexpected role for cytochrome *c* in inhibiting ferroptosis has been demonstrated. Cytochrome *c* increases inositol polyphosphate-4-phosphatase type IA activity, which increases the formation of phosphatidylinositol-3-phosphate, preventing phospholipid peroxidation and plasma membrane rupture. Thus, cytosolic cytochrome *c* complexes govern diverse cell death pathways (promotion of apoptosis and inhibition of ferroptosis) [48].

#### Others

GSH *S*-transferase P1 (GSTP1) detoxifies lipid peroxides by activating selenium-independent GSH peroxidase activity, a mechanism that is both GPX4-independent and FSP1-independent. In addition, SMAD-specific E3 ubiquitin protein ligase 2 promotes GSTP1 ubiquitination and degradation and thereby predisposes cancer cells toward ferroptosis [49].

p53-mediated ferroptosis occurs mainly through ALOX12 lipoxygenase under ROS-induced stress and constitutes another distinct GPX4-independent pathway [50]. iPLA2β, a calcium-independent phospholipase, cleaves acyl tails from the glycerol backbones of lipids and releases oxidized FAs from phospholipids. iPLA2β-mediated lipid detoxification is important for the suppression of p53-mediated ferroptosis, even in GPX4-null cells, suggesting that iPLA2β represses ferroptosis in a GPX4-independent manner [51].

Interleukin-4-induced-1, an amino acid oxidase secreted from immune cells, inhibits ferroptosis by generating indole-3-pyruvate, which scavenges free radicals and activates an antioxidative gene expression [52]. In addition, nitric oxide synthetase 2 suppresses ferroptosis in M1 macrophages by suppressing ALOX15-mediated lipid peroxidation [53].

## Regulation of iron (Fig. 3)

**Fig. 3:**
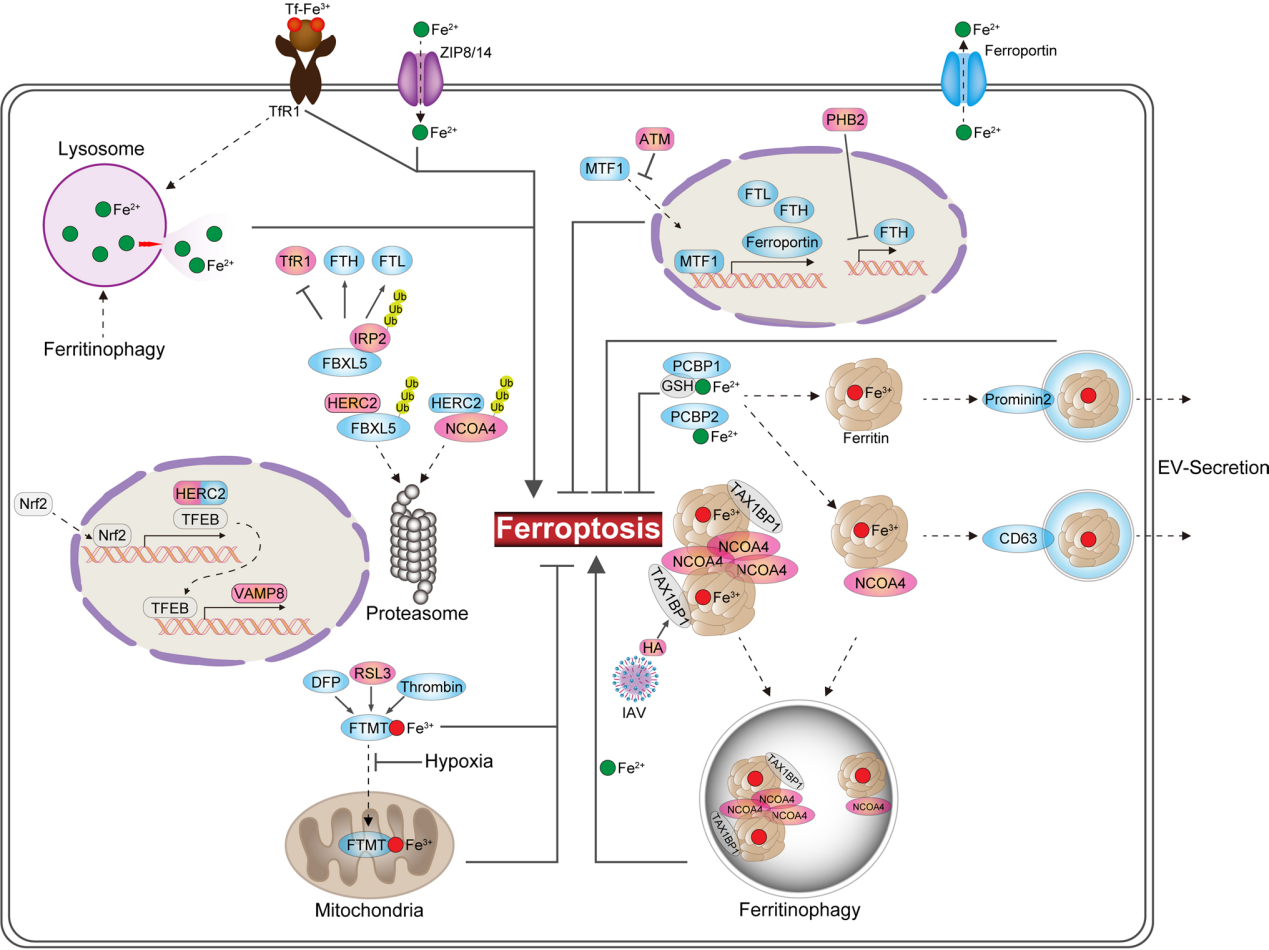
Mechanisms which iron regulates ferroptosis. Ferroptosis is regulated by molecules involved in iron metabolism. Posttranslational modifications of classical iron metabolism-related molecules and changes in ferritin levels significantly influence intracellular iron concentrations, which drive ferroptosis. *ATM* ataxia telangiectasia mutated (DNA damage response serine/threonine kinase), *DFP* deferiprone, *EV* extracellular vesicle, *FBXL5* F-box and leucine-rich repeat protein 5, *FTH* ferritin heavy chain, *FTL* ferritin light chain, *FTMT* mitochondrial ferritin, *HA* hemagglutinin, *HERC2* E3 ubiquitin ligase for NCOA4 and FBXL5, *IAV* influenza virus A, *IRP2* iron regulatory protein 2, *MTF1* metal-regulatory transcription factor 1, *NCOA4* nuclear receptor coactivator 4, *Nrf2* nuclear factor erythroid 2-related factor 2, *PCBP1/2* poly(rC)-binding protein 1/2, *RSL3* RAS-selective lethal compound 3, *TAX1BP1* Tax1-binding protein, *TFEB* transcription factor EB, *TfR* transferrin receptor, *VAMP8* vesicle-associated membrane protein 8, *ZIP8/14* zinc- and iron-related protein 8/14

### Labile iron pool (LIP)

Cellular iron homeostasis is regulated by iron regulatory protein (IRP)1 and IRP2, which control the expression and activities of a series of proteins that are involved in iron import, export, storage, and release [54]. Transferrin [55], transferrin receptor (TfR) [56], Zrt and Irt-like protein 8 (ZIP8, encoded by *SLC39A8*), and ZIP14/*SLC39A14*, which mediates nontransferrin-bound iron uptake by hepatocytes [57], ferroportin [58], and prominin 2 [59], are also involved in the regulation of ferroptosis. In addition to IRPs, the DNA damage response serine/threonine kinase mutated in ataxia telangiectasia also regulates the expression of proteins involved in iron storage [ferritin heavy chain (FTH) and light chain] and export (ferroportin) through the nuclear translocation of metal-regulatory transcription factor 1, thereby regulating the size of the LIP [60].

Cytosolic ferritin and the iron chaperones poly(rC)-binding protein (PCBP)1 and PCBP2 confer resistance to ferroptosis by limiting the availability of labile iron [61]. Conversely, iron can be released from ferritin through the activation of at least two pathways [62]: nuclear receptor coactivator 4 (NCOA4)-mediated ferritin-selective autophagy (ferritinophagy) [63] and NCOA4 condensate-mediated delivery of ferritin to lysosomes via a Tax1-binding protein (TAX1BP1)-dependent noncanonical autophagy pathway [64]. These pathways increase sensitivity to ferroptosis by expanding the LIP [65]. The hemagglutinin of influenza virus A induces ferritin-NCOA4 condensate-mediated ferritinophagy via a TAX1BP1-dependent pathway [66]. Furthermore, we demonstrated that under conditions of iron loading, intracellular ferritin is transferred by NCOA4 to CD63^+^ extracellular vesicles (EVs), which are released into the circulation [62, 67]. These results suggest that CD63^+^ EVs may represent a more potent iron efflux pathway than that mediated by the iron exporter, ferroportin, and thereby suppress cellular sensitivity to ferroptosis, as prominin 2-mediated ferritin-containing multivesicular bodies and exosomes drive resistance to ferroptosis [59, 68].

Two types of ferritin, cytosolic ferritin and mitochondrial ferritin (FTMT), have been identified in a range of organisms, from plants to humans. Although FTMT localizes to the mitochondria via its targeting sequence [69], it reduces the size of the labile iron pool in the cytoplasm and causes a shift of iron from cytosolic ferritin to FTMT [70]. We have also shown in vitro that the extent of the reduction in iron content is greater in the cytosol than in the mitochondria upon the induction of FTMT by deferiprone [71]. FTMT overexpression protects neuroblastoma SH-SY5Y cells from erastin-induced ferroptosis [72] and attenuates cerebral ischemia/reperfusion-induced ferroptosis in mice [73]. FTMT is exclusively present in the cytoplasm under hypoxic conditions because of its cleavage by thrombin [74]. Conversely, the ferroptosis inducer RSL3 increases FTMT expression in the presence of thrombin, suggesting that FTMT expression is induced in ferroptosis as a protective mechanism [74].

Nrf2 also controls the size of the LIP through HECT and RLD domain-containing E3 ubiquitin protein ligase 2 (HERC2) for NCOA4 and F-box and leucine-rich repeat protein 5, and vesicle-associated membrane protein 8 (VAMP8), which mediates autophagosome–lysosome fusion [75]. The deletion of *Nrf2* suppresses *HERC2* expression, leading to an increase in ferritin and NCOA4 expression and the recruitment of apoferritin to autophagosomes. It also reduces *VAMP8* expression through transcription factor EB (TFEB), causing the inhibition of ferritinophagy. Thus, the deletion of *Nrf2* results in an autophagosomal accumulation of apoferritin/NCOA4, an increase in the size of the LIP, and greater sensitivity to ferroptosis.

PCBP1 requires GSH to bind iron [76], suggesting that the availability of labile iron depends on GSH. The coordination of iron and GSH on PCBP1 is required for ferritin binding and iron storage [77]. Thus, depletion of GSH increases the size of the LIP, which in turn increases cellular sensitivity to ferroptosis, but is not essential for ferroptosis, as the direct inhibition or deletion of GPX4 in the presence of GSH is sufficient to induce ferroptosis. Therefore, cysteine deprivation-induced ferroptosis and direct GPX4 inhibition-induced ferroptosis differ with respect to the availability of labile iron.

### Ferroptosis in liver disease (Table 1)

**Table 1:** Roles and mechanisms of ferroptosis in liver diseases

Liver disease	Role of ferroptosis in disease	Activation/inhibition of ferroptosis	Proposed mechanism	Model	References
APAP-induced liver injury	Disease onset	Activation	Increased lipid peroxidation derived from arachidonic acid and autooxidation	Murine	Yamada et al. [78]
	Disease onset	Activation	Mitochondrial dysfunction via VDAC1 oligomerization	Murine	Niu et al. [79]
	Disease restraint	Inhibition	Suppression of ferroptosis via increased 7-DHC by inhibition of DHCR	Murine	Yamada et al. [80]
	Disease onset	Activation	Iron overload in the presence of severe impairment of antioxidant defense mechanism	Murine	Adelusi et al. [81]
Heat stroke-induced acute liver injury	Disease onset	Activation	HO-1-induced activation of NLRP3 inflammasome through promoting PI4P production	Murine	Li et al. [82]
Hepatic IRI	Disease onset	Activation	ACSL4 activation by gp78	Murine	Li et al. [83]
	Disease onset	Activation	Stabilization of ACSL4 through KCNQ1T1/HIF-1α axis	Murine	Zou et al. [84]
	Disease onset	Activation	Downregulation of NADP^+^-dependent malic enzyme through mTOR/SREBP1 signaling suppression	Murine	Fang et al. [85]
	Disease onset	Activation	GPX4 ubiquitination by TMEM16A	Murine	Guo et al. [86]
	Disease onset	Activation	Iron overload	Human	Yamada et al. [87]
	Disease suppression	Inhibition	FTO deficiency-induced posttranscriptional m6A modification of ACSL4 and TfR	Murine	Li et al. [88]
			Increase in 7-DHC by DHCR inhibitor	Murine	Yamada et al. [80]
HBV-induced acute liver failure	Disease onset	Activation	SLC7A11 suppression via EZH2 by HBx protein	Murine	Liu et al. [89]
HCV infection	Inhibition of HCV replication	Activation	Promotion of lipid peroxidation through FADS2-induced conversion of oleic acid to Mead acid and other highly unsaturated fatty acids	Murine	Yamane et al. [91]
	HCC development	Activation	Iron accumulation through ROS-mediated hepcidin suppression	Murine	Nishina et al. [92]
Autoimmune hepatitis	Disease onset	Inactivation	Caveolin protects hepatocytes against ferroptosis in ConA-induced hepatitis by attenuating nitrogen stress	Murine	Deng et al. [93]
	Disease onset	Inactivation	FGF4 inhibits hepatocyte ferroptosis in ConA-induced hepatitis by increasing CISD3 and activating Nrf2/HO-1 signaling	Murine	Jiang et al. [94]
Alcohol-associated liver disease	Disease progression	Activation	Production of acetaldehyde, reduced level of GSH in the mitochondria, ROS production, followed by lipid peroxidation	Murine	Ali et al. [95]
	Disease progression	Activation	Deactivation of GPX4 through GSH exhaustion that is induced by dysregulation of the methionine cycle and transsulfuration pathway	Murine	Luo et al. [96]
	Disease progression	Activation	Iron accumulation related to GCN5L1-modulated CISD1 acetylation and activity	Murine	Yang et al. [97]
	Disease progression	Activation	FNDC3B deletion exacerbates ethanol-induced liver steatosis and lipid peroxidation via AMPK inhibition	Murine	You et al. [98]
MASLD/MASH	Disease progression	Activation	Abnormal hepatic phosphatidylcholine (PC) composition, caused by impaired PUFA synthesis and disorganized FA incorporation into PCs	Murine	Kawamura, et al. [104]
	Disease progression	Activation	Increase in phosphatidylglycerol with two PUFA lipid tails by MUFA and saturated fatty acids	HepG2 cell and murine	Peleman et al. [101]
	Disease repression	Inactivation	Interaction of ferroptotic hepatocytes and neighboring cells such as HSCs and endothelial cells via DAMPs	Murine and endothelial cells	Upchurch et al. [106]
	Disease progression	Activation	Interaction of ferroptotic hepatocytes and neighboring macrophages via DAMPs	Cultured cells	Zanoni et al. [107]
	Disease progression	Activation	Interaction of ferroptotic hepatocytes and neighboring cytotoxic T cells via DAMPs	Cultured cells	Wiernicki et al. [108]
	Disease progression	Activation	Activation of subset of autoaggressive T cells by metabolites such as ATP and acetate	Murine	Dudeck et al. [109]
	Disease progression	Activation	EFHD2 induces proferroptotic factors such as ACSL4 and ALOX12 and malondialdehyde, and downregulates GPX4	Human and murine	Fu et al. [111]
	Disease progression	Activation	Ferritinophagy via TFEB	Murine	Honma et al. [115]
	Disease progression	Activation	Iron accumulation in Kupffer cells through TLR4-dependent hepcidin production by NCF1-induced oxidized phospholipids	Murine	Zhang et al. [116]
	Disease repression	Inactivation	Inhibition of hepatic iron accumulation and c-Myc-ACSL4-triggered ferroptosis by FOT1	Murine	Tao et al. [113]
Liver fibrosis	Disease repression	Activation	Induction of HSC ferroptosis by binding of BECN1 to xCT and blocking of system Xc^−^ activity	Murine	Guo et al. [117]
	Disease repression	Activation	Promotion of HSC ferroptosis via suppression of xCT-driven GPX4 expression by MSC-ex-delivered BECN	Human and murine	Tan et al. [118]
	Disease repression	Activation	Induction of HSC ferroptosis through autophagy signaling by N(6)-methyladenosine modification	Murine	Shen et al. [119]
	Disease repression	Activation	Simvastatin promotes ferroptosis in HSCs by inhibiting the mevalonate pathway without affecting hepatocytes	Human and cultured cells	Kitsugi et al. [120]
	Disease repression	Activation	Sorafenib induces ferritinophagy and ferroptosis in HSCs through downregulating ZFP36	Murine	Zhang et al. [121]
	Disease progression	Inactivation	Inhibition of ferroptosis in HSCs through MafG/MYH9-LCN2 axis	Human and murine	Deng et al. [122]
HCC	HCC development	Activation	Association of ACSL4 with cancer-associated fibroblasts and exhausted tumor immune microenvironment	Human	Toshida et al. [123]
	HCC development	Activation	Promotion of hepatocarcinogenesis by γ-OHPdG	Murine	Fu et al. [124]
	HCC development	Activation	Iron overload induces HCC development through the accumulation of lipid peroxidation products and oxidative DNA damage	Murine	Furutani et al. [125]
	Suppression of HCC development	Inhibition	SLC7A11 induction by ATF4	Murine	He et al. [126]
	Tumor promotion	Inhibition	Upregulation of NQO1, GPX4, SLC7A11, FTH, metallothionein 1G, and TIGAR through Keap1 inactivation-mediated repression of Nrf2 degradation	Cultured cells and human	Sun et al. [127] Yang et al. [128] Toshida et al. [129]
	Tumor suppression	Activation	APE1-mediated reduction of Nrf2 through Akt oxidation, resulting in decreased expression of SLC7A11 and GPX4	Cultured cells and murine	Du et al. [130]
	Tumor promotion	Inhibition	Prevention from Nrf2 degradation through Keap1 inactivation mediated by interaction of mitochondrial TSPO with p62	Cultured cells	Zhang et al. [131]
	Immune evasion	Inhibition	TSPO-mediated upregulation of PD-L1 through Nrf2 activation	Murine	Zhang et al. [131]
	Immune evasion	Inhibition	Upregulation of PD-L1 expression in CD8^+^ T cells and MDSCs infiltration mediated by GPX4 loss-mediated ferroptotic hepatocyte death	Murine	Chonche et al. [132]
	Resistance to sorafenib	Inhibition	Inhibition of ferroptosis by ABCC5 through activation of PI3K/AKT/Nrf2	Cultured cells	Huang et al. [136]
	Resistance to sorafenib	Inhibition	Downregulation of GSTZ1 which inhibits Nrf2 expression	Cultured cells and murine	Wang et al. [137]
	Resistance to sorafenib	Inhibition	QSOX1-promoted EGFR degradation and its intracellular endosomal trafficking, leading to Nrf2 suppression	Human and murine	Sun et al. [138]
	Tumor promotion	Inhibition	S100P-mediated lysosomal degradation of ACC1 via p62-dependent selective autophagy	Cultured cells	Yang et al. [139]
	Tumor promotion	Inhibition	ATP production and deactivation of AMPK by MCT1-mediated lactate uptake upregulates SREBP1 and SCD1 to promote MUFAs production	Cultured cells	Zhao et al. [140]
	Tumor promotion	Inhibition	Decrease in FCN3 increases MUFA levels through upregulation SREBP1c by stabilizing IR-β	Murine	Yuan et al. [141]
	Tumor promotion	Inhibition	Lower GLS2 mRNA levels suppress ROS production by reducing the conversion of glutamate to α-ketoglutarate	Human and murine	Suzuki et al. [142]
	Tumor promotion	Low to moderate activation	Hyaluronic acid fragments and oxidized phospholipids released by ferroptotic cells promote IL-1β release from macrophages to trigger neutrophil-mediated sinusoidal vascular remodeling	Human and murine	Mu et al. [143]
	Resistance to sorafenib	Inhibition	TEAD1-dependent and ATF4-mediated SLC7A11 induction through YAP/TAZ signaling	Cultured cells and murine	Gao et al. [144]
	Tumor promotion	Inhibition	Upregulation of LCN2 by LIFR loss depletes iron, leading to resistance to ferroptosis	Murine	Yao et al. [145]
	Tumor promotion	Inhibition	SLC7A11/GPX4 induction through HBx protein-mediated HSPA8 upregulation	Murine	Wang et al. [146]
	Tumor suppression	Activation	Blocking of HSPA8 transcription by LACTB results in activation of ferritinophagy and inhibition of SLC7A11/GPX signaling. LACTB is a downstream effector of lenvatinib	Murine	Zeng et al. [147]
	Tumor suppression	Activation	p-53-induced PCDHB14 downregulates SLC7A11 expression via p65 ubiquitination	Murine	Liu et al. [148]
	Tumor promotion	Inhibition	Suppression of p53-mediated ferroptosis by ZNF498 via reduction of p53 Ser46 phosphorylation	Human and murine	Zhang et al. [149]
	Tumor suppression	Activation	SLC7A11/SLC3A2 downregulation by IFN-γ released from CD8^+^ T cells during cancer immunotherapy	Murine	Wang et al. [150]

*ABCC5* ATP-binding cassette subfamily C member 5, *ACC1* acetyl-CoA carboxylase 1, *ACSL4* acyl-coenzyme A synthetase long-chain family member 4, *AKT* serine/threonine kinase, *ALOX12* arachidonate 12-lipoxygenase, *AMPK* AMP-activated protein kinase, *APAP* acetaminophen, *BECN1* Beclin 1, *CISD3* CDGSH-iron-sulfur domain-containing protein 3, *ConA* concanavalin A, *DAMPs* damage-associated molecular patterns, *7DHC* 7-dehydrocholesterol, *DHCR* dehydrocholesterol reductase, *EGFR* epidermal growth factor receptor, *EFHD2* EF-hand domain family member D2, *EZH2* zeste homolog, *FA* fatty acid, *FADS2* fatty acid desaturase 2, *FCN3* ficolin 3, *FGF4* fibroblast growth factor 4, *FNDC3B* fibrinogen type III domain-containing protein 3B, *FOT1* FerroTerminator 1, *FTH* ferritin heavy chain, *FTO* fat mass and obesity-associated gene, *γ-OHPdG* γ-hydroxy-1,N^2^-propanodeoxyguanosine, *GCN5L1* amino acid synthesis 5-like 1, *gp78* glycoprotein 78, *GPX4* glutathione peroxidase 4, *GSH* glutathione, *GSTZ1* glutathione S-transferase zeta 1, *HBV* hepatitis B virus, *HBx* hepatitis B x, *HCC* hepatocellular carcinoma, *HCV* hepatitis C virus, *HIF-1α* hypoxia inducible factor 1α, *HO-1* Heme oxygenase 1, *HSC* hepatic stellate cell, *HSPA8* Heat shock protein family A member 8, *IR-β* insulin receptor β, *IRI* ischemia–reperfusion injury, *KCNQ1T1* KCNQ1 overlapping transcript 1, *LACTB* serine beta-lactamase-like protein, *LCN2* Lipocalin 2, *LIFR* leukemia inhibitory factor receptor, *m6A* N6-methyladenosine, *MafG* Maf bZIP transcription factor G, *MASH* metabolic dysfunction-associated steatohepatitis, *MASLD* metabolic dysfunction-associated steatotic liver disease, *MCT1* monocarboxylate transporter 1, *MSC-exs* exosomes secreted from human mesenchymal stem cells, *mTOR* mammalian target of rapamycin, *MUFA* monounsaturated fatty acid, *MYH9* nonmuscle myosin heavy chain IIa, *NCF1* neutrophil cytosolic factor 1, *NLRP3* NOD-like receptor family pyrin domain-containing 3, *NQO1* NAD(P)H quinone dehydrogenase, *Nrf2* nuclear factor erythroid 2-related factor 2, *PCDHB14* protocadherin beta-14, *PD-L1* programmed death Ligand 1, *PI3K* phosphatidylinositol 3-kinase, *PI4P* phosphatidylinositol 4-phosphate, *PUFA* polyunsaturated fatty acid, *QSOX1* quiescin-sulfhydryl oxidase 1, *ROS* reactive oxygen species, *S100P* S100 calcium-binding protein P, *SCD1* stearoyl-CoA desaturase, *SLC3A2* solute carrier family 3 member 2, *SLC7A11* solute carrier family 7 member 11, *SREBP1* sterol regulatory element-binding protein 1, *TAZ* transcriptional coactivator with PDZ-binding motif, *TEAD1* TEA domain family member 1, *TFEB* transcription factor EB, *TfR* transferrin receptor, *TIGAR TP53*-induced glycolysis and apoptosis regulator, *TLR4* Toll-like receptor 4, *TMEM16A* transmembrane member 16A, *TSPO* translocator protein, *VDAC1* voltage-dependent anion channel 1, *xCT* SLC7A11, *YAP* Yes-associated protein, *ZFP36* zinc-finger protein 36 homolog, *ZNF498* zinc-finger protein

The liver plays a central role in systemic iron homeostasis, and a number of hepatic metabolic pathways are linked to the occurrence and regulation of ferroptosis. Consequently, hepatic disorders in liver diseases can be critical triggers of ferroptotic cell death. Given this unique physiological context, increasing evidence suggests a strong association between ferroptosis and the pathogenesis of liver diseases. In the following sections, we provide an overview of the current understanding of how ferroptosis contributes to the development and progression of liver diseases and potential therapeutic strategies against ferroptosis.

## Acute liver injury

### Acetaminophen (APAP)-induced hepatocellular death

Previous studies have suggested a critical role for ferroptosis in APAP-induced hepatocellular death [78]. APAP overdose induces ferroptosis through mitochondrial dysfunction via voltage-dependent anion channel 1 (VDAC1) oligomerization. Protecting mitochondria via inhibition of VDAC1 oligomerization attenuates ferroptosis by restoring ceramide and cardiolipin contents in APAP-induced liver injury [79]. Inhibition of 7-dehydrocholesterol reductase (DHCR7) increases its substrate 7-DHC, which suppresses ferroptosis, and genetic ablation of *Dhcr7* has been reported to prevent APAP-induced acute liver failure [80]. Another study revealed that ferroptosis does not contribute to APAP-induced cell death under normal circumstances but rather contributes to lipid peroxidation under conditions of iron overload in the context of severe impairment of antioxidant defense mechanisms [81]. Thus, there is still controversy regarding roles of ferroptosis in APAP-induced liver injury.

### Heat stroke-induced acute liver injury

Acute liver injury is a frequently documented complication of Heat stroke, and is uniquely caused by cellular thermotoxic effects and intense inflammatory responses. Heat-induced heme oxygenase 1 (HO-1) mediates ferroptosis in Kupffer cells and promotes Hepatic cell death through NOD-like receptor family pyrin domain-containing 3 (NLRP3) inflammasome activation and IL-1β in heat stroke mice. HO-1 facilitates NLRP3 inflammasome activation by promoting lipid phosphatidylinositol 4-phosphate production [82].

### Ischemia–reperfusion injury (IRI)-induced acute liver injury

Hepatic IRI-induced acute liver injury occurs in a wide range of clinical contexts, including liver transplantation, systemic shock, heart failure, hemorrhage, and sepsis. A complex mixture of types of cell death, including apoptosis, necroptosis, and ferroptosis, contributes to hepatic IRI. Many pathways have been implicated in the ferroptosis that occurs during hepatic IRI, including an increase in ACSL4 expression induced by gp78, an E3 ubiquitin ligase [83], increased stability of ACSL4, secondary to the transcription of KCNQ1 overlapping transcript 1 induced by hypoxia inducible factor 1α [84], downregulation of NADP^+^-dependent malic enzyme mediated by mTOR/SREBP1 suppression [85], and GPX4 ubiquitination by transmembrane member 16A, a component of the hepatocyte Ca^2+^-activated chloride channel [86]. High serum iron concentrations in Liver donors are associated with a significantly increased risk of Liver damage in transplantation recipients, and ferroptosis inhibitors, such as ferrostatin 1 and deferoxamine, have also been shown to prevent hepatic IRI in a murine model [87]. The expression of fat mass and obesity-associated gene (FTO), an N^6^-methyladenosine (m^6^A) demethylase, is downregulated in hepatic IRI. FTO deficiency promotes the posttranscriptional m^6^A modification of ACSL4 and TfR and induces their expression. Nicotinamide mononucleotide increases FTO demethylase activity, which leads to the suppression of ferroptosis in hepatic IRI [88]. The DHCR inhibitor, AY9944, which increases 7-DHC, inhibits hepatic IRI by suppressing ferroptosis [80].

### Hepatitis B virus (HBV)-induced acute liver failure

HBV is the principal cause of acute liver failure worldwide. HBV X protein (HBx), an essential regulatory protein, plays an important role in the development of HBV-associated Liver disease. HBx facilitates ferroptosis in D-galactosamine-induced acute Liver failure by suppressing enhancer of zeste homolog 2-mediated SLC7A11 expression [89].

## Chronic liver injury

### Hepatitis C virus (HCV) infection

HCV is unique in that its replicative activity is regulated by lipid peroxidation within replicase membranes derived from the endoplasmic reticulum. Membrane-proximal regions of the NS3/4A protease and NS5B RNA-dependent RNA polymerase act as lipid peroxidation sensors that reduce replicase activity through conformational changes caused by lipid peroxidation. This lipid peroxidation-sensitive phenotype of HCV is associated with lower replicative activity and its greater persistence in damaged liver tissue [90]. Fatty acid desaturase 2, which converts oleic acid to Mead acid and other PUFAs, sensitizes cells to ferroptosis and restricts HCV replication by promoting lipid peroxidation [91]. We have previously shown that HCV induces hepatic iron accumulation through the ROS-mediated repression of hepcidin transcription [92]. Because PUFAs are oxidized in an iron-dependent manner, hepatic iron overload alongside HCV infection may induce ferroptosis and affect HCV replication.

### Autoimmune hepatitis

Autoimmune hepatitis is a chronic inflammatory liver disease that can lead to cirrhosis and liver failure. The concanavalin A-induced liver injury mouse model is a typical animal model that focuses on T-cell-dependent hepatic damage in the field of research on autoimmune hepatitis.

Caveolin-1 deficiency exacerbates concanavalin A-induced hepatocellular ferroptosis associated with excessive nitrogen response. Additionally, immune inhibition by gadolinium chloride, which depletes Kupffer cells, increases hepatic caveolin-1 but inhibits ferroptosis and nitrative stress under concanavalin A exposure. Downstream of caveolin-1, reactive nitrogen species-mediated ferroptosis is a pivotal step that drives the execution of acute immune-mediated hepatic damage [93].

Hepatic fibroblast growth factor 4 (FGF4) depletion increases susceptibility to lipid peroxidation and iron accumulation and to hepatic lesions and inflammation caused by concanavalin A administration. FGF4 plays a protective role in autoimmune hepatitis progression, with FGF4 treatment inhibiting the ferroptosis of Hepatocytes by increasing CDGSH iron-sulfur domain-containing protein 3 (CISD3) and activating Nrf2/HO-1 signaling [94].

### Alcohol-associated liver disease

Iron loading is one of the characteristic features of alcohol-associated liver disease. Hepcidin is a liver-specific iron hormone that limits intestinal iron absorption and iron release from macrophages. Alcohol-induced suppression of hepcidin is the main cause of systemic iron loading in alcohol consumers. Alcohol metabolism generates a large amount of acetaldehyde, reduces the levels of GSH in the mitochondria, and increases ROS production, followed by elevated lipid peroxidation in liver cells [95]. This pathophysiology provides a strong explanation for ferroptosis initiation in the livers of patients with alcohol-associated liver disease. In fact, long-term ethanol feeding has been shown to induce ferroptosis in mice, as evidenced by the increased expression of ferroptosis-related genes, such as CD36, ACSL4, and POR, along with lipid peroxidation, and labile iron accumulation in the liver. In addition, long-term ethanol feeding deactivates GPX4 through GSH exhaustion, which is induced by dysregulation of the methionine cycle and transsulfuration pathway [96].

Gene Expression Omnibus dataset analysis revealed that general control of amino acid synthesis 5-like 1 (GCN5L1) is negatively associated with the progression of alcohol-associated liver disease. GCN5L1 targets CISD1, which regulates both mitochondrial iron transport into the matrix and mitochondrial respiratory capacity. Thus, GCN5L1-modulated CISD1 acetylation and activity are crucial in preventing iron accumulation and ferroptosis in response to alcohol exposure [97].

Alcohol exposure increases fibronectin type III domain-containing protein 3B (FNDC3B) in cultured cells and mouse livers. FNDC3B deletion exacerbates ethanol-induced liver steatosis and lipid peroxidation via AMPK inhibition. Thus, FNDC3B plays a critical role in preventing hepatic steatosis and ferroptosis in response to chronic alcohol consumption [98].

### Metabolic dysfunction-associated steatotic liver disease (MASLD)

Nonalcoholic fatty liver disease (NAFLD) has rapidly become the most common chronic Liver disease globally and is currently estimated to affect up to 38% of the global adult population [99]. Recently, it was proposed that the term NAFLD be replaced by MASLD and that the term nonalcoholic steatohepatitis (NASH) be replaced by metabolic dysfunction-associated steatohepatitis (MASH) [100]. In the present review, we use MASLD and MASH according to the latest nomenclature. A proportion of biopsy-proven MASLD patients exhibit hepatic ferroptosis and lower GPX4 levels [101]. Hepatic ferroptosis, rather than necroptosis, has been reported to play an important role in the transition from simple steatosis to MASH [102]. In addition, ferroptosis and lipid metabolic disorders play critical roles in MASH progression, and inhibiting ferroptosis significantly reduces the severity of MASH [103]. Abnormal hepatic phosphatidylcholine composition, caused by impaired PUFA synthesis and disorganized FA incorporation into phosphatidylcholines, has been identified in a genetic murine model of MASH that shows progressive liver injury and hepatic carcinogenesis [104]. Unexpectedly, MUFA and saturated fatty acid supplementation has been shown to increase the level of phosphatidylglycerol with two PUFA lipid tails and maintain ferroptosis sensitivity [101].

Ferroptotic hepatocytes release many DAMPs, such as oxidized PLs and oxidized lipids, in the MASLD microenvironment [105]. These DAMPs promote histological aspects of MASLD, including disease activity and fibrosis, through the interaction of ferroptotic hepatocytes with neighboring cells, such as hepatic stellate cells (HSCs) and endothelial cells [106], macrophages [107], cytotoxic T cells [108], or myofibroblasts. Metabolites, such as ATP and acetate, can activate subsets of autoaggressive T cells recently discovered in MASLD [109].

MASH liver demonstrates a significant shift in immune composition, including Kupffer cell depletion/transition, and the appearance of monocyte-derived macrophages and MASH-associated macrophages [110]. EF-hand domain family member D2 (EFHD2) regulates the replacement of resident Kupffer cells by infiltrating monocytes during MASH. EFHD2 in myeloid cells mediates interferon-γ signaling, which triggers immune and inflammatory responses in macrophages during MASH. *Efhd2* deletion improves hepatic steatosis, reduces immune cell infiltration, and inhibits ferroptosis through the attenuation of proferroptotic factors (ACSL4 and ALOX12) and the lipid peroxidation product malondialdehyde and the upregulation of GPX4 in MASH model mice. Thus, EFHD2 promotes ferroptosis in MASH [111].

Iron overload has been demonstrated in large cohort studies of MAFLD patients [112]. MAFLD patients exhibit hepatic iron excess which is strongly positively correlated with disease progression [113]. Greater Duodenal iron absorption via upregulated divalent metal transporter 1 (DMT1) has been shown in MASH patients. Furthermore, an unidentified humoral factor(s) in the sera of MASH patients was shown to activate IRP1, which increased DMT1 expression through the IRP/iron-response element system [114]. A recent study revealed that iron overload promotes ferritinophagy via the nuclear translocation of TFEB, which exacerbates MASH in mice [115]. Interestingly, not only Hepatocytes but also Kupffer cells are sensitive to ferroptosis induced by iron accumulation in MASH patients. The impaired self-renewal of Kupffer cells triggers inflammation in MASH, and neutrophil cytosolic factor 1 (NCF1) is upregulated in Kupffer cells in MASH patients and mice. Large amounts of oxidized phospholipids, the production of which is induced by macrophage NCF1, promote Toll-like receptor 4-dependent hepcidin production in hepatocytes, leading to greater iron deposition and subsequent ferroptosis in Kupffer cells [116]. The mRNA levels of both *c-Myc* and *ACSL4* in the liver are significantly higher in patients with MASH than in those with MAFLD. FerroTerminator-1 concurrently inhibits hepatic iron accumulation and c-Myc-ACSL4-triggered ferroptosis and consequently reverses liver injury in various MASH models [113].

### Liver fibrosis

HSCs play a critical role in liver fibrosis. Activated HSCs secrete excessive amounts of profibrogenic factors and extracellular matrix that form the basis of liver fibrosis. Ferroptosis in hepatocytes exerts a pathological effect and inhibition of ferroptosis in hepatocytes is therapeutic in nonneoplastic liver conditions, whereas ferroptosis in HSCs has the opposite effect. Therefore, the promotion of ferroptosis in HSCs can be a therapeutic tool for preventing liver fibrosis.

Beclin 1 induces ferroptosis through binding to xCT and blocking X_c_^−^ activity [117]. Beclin 1 which is delivered from exosomes secreted by human mesenchymal stem cells promotes HSC ferroptosis by suppressing xCT-driven GPX4 expression and consequently suppresses collagen deposition in experimental mouse fibrotic livers [118]. The m^6^A modification serves as the most abundant posttranscriptional mechanism in eukaryotic mRNAs. The m^6^A reader YTHDF1 promotes the stability of Beclin 1 mRNA via recognition of the m^6^A-binding site, thus triggering autophagy activation and eventually leading to HSC ferroptosis. These results identify m^6^A modification-dependent ferroptosis as a potential target for the treatment of liver fibrosis [119]. Simvastatin, a 3-hydroxy-3-methylglutaryl-coenzyme A reductase inhibitor, inhibits HSC activation, which is accompanied by iron accumulation, oxidative stress, and lipid peroxidation, and reduces GPX4 expression. These results indicate that simvastatin promotes ferroptosis in HSCs by inhibiting the mevalonate pathway. Notably, statins downregulate the expression of GPX4 in HSCs without affecting hepatocytes in human tissue samples [120].

The RNA-binding protein, zinc-finger protein 36 homolog (ZFP36) suppresses ferroptosis through deactivation of autophagy in HSCs. Importantly, sorafenib, a multikinase inhibitor approved for the treatment of HCC, downregulates ZFP36, activates ferritinophagy, and induces ferroptosis in human HSCs [121]. Maf bZIP transcription factor G (MafG) is upregulated in human and murine liver fibrosis. MafG knockdown increases HSC ferroptosis. MafG physically interacts with nonmuscle myosin heavy chain IIa (MYH9) to transcriptionally activate the expression of Lipocalin 2 (LCN2), a known suppressor for ferroptosis. Thus, the MafG/MYH9–LCN2 signaling pathway could be a novel target for the treatment of liver fibrosis [122].

## HCC

The role of ferroptosis in HCC has at least two implications: it promotes HCC development under nonneoplastic liver conditions and has therapeutic potential for HCC, as liver cancer cells display intrinsic or acquired resistance to ferroptosis. Thus, ferroptosis is a promising target for the prevention and treatment of HCC. In contrast to various cancers, ACSL4, a ferroptosis driver, in HCC has been reported to be a poor prognostic factor through its association with cancer-associated fibroblasts and exhausted tumor immune microenvironment [123].

Lipid peroxidation-derived mutagenic DNA adducts such as γ-hydroxy-1,N^2^-propanodeoxyguanosine (γ-OHPdG) promote hepatocarcinogenesis in mice, and higher levels of γ-OHPdG are associated with poor survival and recurrence-free survival in HCC patients [124]. We previously showed that iron overload induces HCC development through the accumulation of lipid peroxidation products and oxidative DNA damage in transgenic mice expressing the HCV polyprotein [125]. ATF4 inhibits ferroptosis-dependent inflammatory cell death, which is associated with compensatory proliferation and hepatocarcinogenesis. Mechanistically, ATF4 suppresses hepatocarcinogenesis by inducing SLC7A11, which inhibits stress-related ferroptosis. [126].

### Regulatory mechanisms of ferroptosis in HCC

#### Nrf2 pathway

HCC cells suppress Nrf2 degradation through p62-mediated Kelch-like ECH-associated protein 1 inactivation, which leads to Nrf2 nuclear translocation. Nrf2-mediated ferroptosis resistance is achieved by increases in the expression of antioxidant or detoxification genes, including those encoding *NAD(P)H quinone dehydrogenase* (*NQO1*), *GPX4*, *SLC7A11*, *FTH*, *metallothionein 1G*, and *TP53-induced glycolysis and apoptosis regulator* [127–129]. The inhibition of apurinic/apyrimidinic endonuclease 1 reduces Nrf2 expression through Akt oxidation in HCC, resulting in ferroptosis through decreased expression of SLC7A11 and GPX4 [130]. Mitochondrial translocator protein (TSPO) promotes the progression of HCC through the p62-mediated prevention of the proteasomal degradation of Nrf2. Furthermore, TSPO reinforces the immunological escape of HCC cells by increasing programmed death Ligand 1 (PD-L1) expression through increased Nrf2-mediated transcription [131]. Surprisingly, hepatocyte-specific GPX4 deletion does not suppress hepatocellular tumorigenesis. Instead, GPX4-associated ferroptotic hepatocyte death causes a tumor-suppressing immune response that is characterized by a CXCL10-dependent infiltration of CD8^+^ T cell that is counterbalanced by PD-L1 upregulation on tumor cells and high-mobility group box 1-mediated infiltration of myeloid-derived suppressor cells [132].

Sorafenib induces ferroptosis by inhibiting SLC7A11 [133]. Lenvatinib, another multikinase inhibitor, induces ferroptosis by inhibiting fibroblast growth factor receptor-4, which suppresses SLC7A11 [134]. Sorafenib also facilitates FSP1 ubiquitination through the ERK pathway, thereby inducing ferroptosis in HCC cells [135]. ATP-binding cassette subfamily C member 5, a member of the ATP-binding cassette protein family, whose expression is markedly induced in sorafenib-resistant HCC cells, inhibits ferroptosis through activation of the PI3K/AKT/Nrf2 axis [136]. Glutathione S-transferase zeta 1 (GSTZ1) inhibits Nrf2 expression and increases sorafenib-induced ferroptosis, whereas sorafenib-resistant HCC cells have low GSTZ1 expression [137]. Quiescin-sulfhydryl oxidase 1 promotes the ubiquitin-mediated degradation of EGFR and accelerates its intracellular endosomal trafficking, leading to the suppression of Nrf2 activity, which facilitates sorafenib-induced ferroptosis [138]. Thus, Nrf2 activation is associated with resistance to sorafenib-induced ferroptosis in HCC cells.

#### Other pathways

While the Nrf2 pathway has been extensively studied as a critical regulator of ferroptosis in HCC, emerging evidence suggests that a variety of alternative pathways have also been implicated in modulating ferroptosis in HCC.

##### S100 calcium-binding protein P (S100P)

S100P is significantly upregulated in ferroptosis-resistant HCC cells. S100P facilitates lysosomal degradation of acetyl-CoA carboxylase (ACC1), which is required for de novo biosynthesis of lipids. S100P-mediated ACC1 degradation relies on Ras-related protein Rab-5C (RAB5C), which directs ACC1 to lysosomes via p62-dependent selective autophagy. Thus, S100P functions as a ferroptosis suppressor to promote HCC development by rewriting lipid metabolism [139].

##### MUFA production

Lactate-rich Liver cancer cells exhibit increased resistance to ferroptotic damage. Monocarboxylate transporter 1 (MCT1)-mediated lactate uptake facilitates ATP production in HCC cells and deactivates AMPK, leading to the upregulation of SREBP1 and downstream stearoyl-CoA desaturase-1 (SCD1) to promote the production of antiferroptotic MuFAs. Blocking lactate uptake via hydroxycarboxylic acid receptor 1/MCT1 inhibition facilitates ferroptosis by downregulating SCD1, which may synergize with its ACSL4-promoting effect [140]. The observation that AMPK deactivation confers ferroptosis resistance contradicts findings in an alcoholic liver disease model, where FNDC3B deletion promotes ferroptosis via AMPK inhibition. This discrepancy is likely attributable to the differences in disease context, such as neoplastic versus nonneoplastic liver conditions.

A recent study demonstrated that reduced expression of ficolin 3, a component of the complement system, leads to MuFA accumulation in human HCC and thereby promotes ferroptosis resistance. Mechanistically, ficolin 3 directly binds to the insulin receptor β (IR-β) and its pro-form, resulting in IR-β inactivation. The inactivation of IR-β reduces the expression of SREBP1c, which subsequently suppresses the transcription of genes related to de novo lipogenesis and lipid desaturation and consequently downregulates MUFA levels to promote ferroptosis [141].

##### Glutamine synthetase 2 (GLS2)

The Cancer Genome Atlas database revealed that the mRNA levels of GLS2 are lower in human HCC tissues than in normal liver tissues. GLS2 increases lipid ROS production by facilitating the conversion of glutamate to α-ketoglutarate, thereby promoting ferroptosis. The reduced expression of GLS2 is likely due to the hypermethylation of CpG islands in the *Gls2* promoter, which in turn results in the downregulation of *Gls2* mRNA expression [142].

##### Ferroptosis-driven inflammation

HCC patients with low-to-moderate activation of ferroptosis in tumors have a greater risk of recurrence than those with no or high activation of ferroptosis. Macrophages selectively accumulate in areas with a significant number of ferroptotic cells, and secrete proinflammatory IL-1β to trigger neutrophil-mediated sinusoidal vascular remodeling, thereby creating a favorable microenvironment for aggressive tumor growth. Hyaluronic acid fragments released by cancer cells upregulate the NLRP3 inflammasome in macrophages in an NF-κB-dependent manner, and oxidized phospholipids secreted by ferroptotic cells activate the NLRP3 inflammasome to release functional IL-1β. Targeting the ferroptosis-induced inflammatory axis increases the efficacy of sorafenib [143].

##### YAP/transcriptional coactivator with PDZ-binding motif (TAZ)

The YAP/TAZ transcription factors promote sorafenib resistance by suppressing ferroptosis in HCC cell lines. The YAP/TAZ transcription factors increase SLC7A11 in a TEA domain family member 1-dependent manner and sustain the stability, nuclear localization, and transcriptional activity of ATF4, which cooperates with SLC7A11 induction [144].

##### Leukemia inhibitory factor receptor (LIFR)/LCN2 axis

LIFR, a negative regulator of NF-κB signaling and LCN2, is expressed at low levels in HCC cells. LIFR loss upregulates LCN2, which depletes iron, leading to resistance to ferroptosis [145].

##### p53-centered regulatory pathways of ferroptosis

Heat shock protein A8 (HSPA8) is upregulated by the HBx protein, which in turn suppresses ferroptosis by increasing the expression of SLC7A11 and GPX4 [146]. Interestingly, the HBx protein can also promote ferroptosis in an acute liver injury mouse model, suggesting that the function of HSPA8 in ferroptosis may be context dependent. These findings highlight HSPA8 as a potential therapeutic target for selectively inducing ferroptosis in HCC. Serine beta-lactamase-like protein (LACTB), a mitochondrial serine protease, represses HSPA8 transcription in a p53-dependent manner, resulting in the activation of NCOA4-mediated ferritinophagy and the inhibition of SLC7A11/GPX signaling, thereby inhibiting liver cancer progression through ferroptosis [147]. Notably, LACTB is also a downstream effector of lenvatinib and the efficacy of lenvatinib is potentiated by LACTB in liver cancer.

p53-induced protocadherin beta 14, a member of the cadherin superfamily, downregulates the expression of SLC7A11 via degradation of p65, a transcription factor of SLC7A11 [148]. In contrast, the zinc-finger protein ZNF498 promotes liver cancer cell growth by suppressing p53-mediated apoptosis and ferroptosis by reducing the Ser46 phosphorylation of p53 [149]. Collectively, these findings define p53-mediated pathways that modulate ferroptosis in HCC and represent promising targets for therapeutic intervention.

#### Tumor microenvironment

Interferon-γ released from CD8^+^ T cells activated by immunotherapy reduces the expression of SLC7A11 and SLC3A2, impairs cystine uptake by tumor cells, and consequently promotes ferroptosis in tumors during cancer immunotherapy [150]. Because sorafenib also inhibits the expression of SLC7A11, the combination of sorafenib and PD-L1 inhibitors might cooperatively induce ferroptosis. Conversely, increasing evidence suggest that CD8^+^ T cells, tumor-associated macrophages, and other immune subsets undergo ferroptosis under the influence of oxidative and iron-rich tumor microenvironment, which can modify antitumor immunity.

## Concluding remarks and future perspectives

Considerable progress has been made with respect to our understanding of the status of ferroptosis as a promising target for the prevention and treatment of many forms of liver disease through the identification of critical regulators of ferroptosis. However, just as iron can be both essential and toxic for organisms, ferroptosis is a double-edged sword, because it promotes hepatocarcinogenesis on the one hand and suppresses tumor growth on the other hand. Liver cancer cells have intrinsic or acquired resistance to ferroptosis, which is mediated via a complex mechanism involving various antioxidant systems. Therefore, a fuller understanding of the complex regulation of ferroptosis is needed, so that ferroptosis can be used as a therapeutic target for liver diseases.

## Abbreviations

ACC1: Acetyl-CoA carboxylase 1
ACSL4: Acyl-coenzyme A synthetase long-chain family member 4
AKT: Serine/threonine kinase
ALOX: Arachidonate lipoxygenase
AMPK: AMP-activated protein kinase
APAP: Acetaminophen
ATF4: Activating transcription factor 4
BH_4_: Tetrahydrobiopterin
CEPT1: Choline/ethanolamine phosphotransferase 1
CISD: CDGSH-iron-sulfur domain-containing protein
CoQ_10_: Coenzyme Q_10_
DAMP: Damage-associated molecular pattern
DHCR7: 7-Dehydrocholesterol reductase
DMT1: Divalent metal transporter 1
EFHD2: EF-hand domain family member D2
EV: Extracellular vesicle
FA: Fatty acid
FFAR2: Free fatty acid receptor 2
FGF4: Fibroblast growth factor 4
FNDC3B: Fibronectin type III domain-containing protein 3B
FSP1: Ferroptosis suppressor protein 1
FTH: Ferritin heavy chain
FTMT: Mitochondrial ferritin
FTO: Fat mass and obesity-associated gene
GCN5L1: General control of amino acid synthesis 5-like 1
GLS2: Glutamine synthetase 2
GPX4: Glutathione peroxidase 4
GSH: Glutathione
GSTP1: Glutathione *S*-transferase P1
GSTZ1: Glutathione S-transferase zeta 1
γ-OHPdG: γ-Hydroxy-1,N^2^-propanodeoxyguanosine
HBV: Hepatitis B virus
HBx: HBV X protein
HERC2: HECT and RLD domain-containing E3 ubiquitin protein ligase 2
HPCAL1: Hippocalcin-like 1
HO-1: Heme oxygenase 1
HSPA8: Heat shock protein A8
IRI: Ischemia–reperfusion injury
IRP: Iron regulatory protein
LACTB: Serine beta-lactamase-like protein
LCN2: Lipocalin 2
LIFR: Leukemia inhibitory factor receptor
LIP: Labile iron pool
LPCAT3: Lysophosphatidylcholine acyltransferase 3
MafG: Maf bZIP transcription factor G
MASH: Metabolic dysfunction-associated steatohepatitis
MASLD: Metabolic dysfunction-associated steatotic liver disease
MBOAT1: Membrane-bound O-acyltransferase domain-containing 1
MCT1: Monocarboxylate transporter 1
mTOR: Mammalian target of rapamycin
MUFA: Monounsaturated fatty acid
MYH9: Nonmuscle myosin heavy chain IIa
m^6^A: N^6^-Methyladenosine
NADPH: Nicotinamide adenine dinucleotide phosphate
NAFLD: Nonalcoholic fatty liver disease
NASH: Nonalcoholic steatohepatitis
NCF1: Neutrophil cytosolic factor 1
NCOA4: Nuclear receptor coactivator 4
NLRP3: NOD-like receptor family pyrin domain-containing 3
Nrf2: Nuclear factor erythroid 2-related factor 2
PCBP1/2: Poly-(rC)-binding protein ½
PD-L1: Programmed death ligand 1
PI3K: Phosphatidylinositol 3-kinase
PL: Phospholipid
POR: P450 oxidoreductase
PRDX6: Peroxiredoxin 6
PUFA: Polyunsaturated fatty acid
RSL3: RAS-selective lethal compound 3
ROS: Reactive oxygen species
SCD1: Stearoyl-CoA desaturase
SCLY: Selenocysteine lyase
SLC7A11: Solute carrier family 7 member 11
SREBP1: Sterol regulatory element-binding protein 1
TAX1BP1: Tax1-binding protein
TAZ: Transcriptional coactivator with PDZ-binding motif
TfR: Transferrin receptor
TSPO: Translocator protein
VAMP8: Vesicle-associated membrane protein 8
VDAC1: Voltage-dependent anion channel 1
YAP: Yes-associated protein
ZFP36: Zinc-finger protein 36 homolog
7-DHC: 7-Dehydrocholesterol
